# The enhancing effect of *Acanthopanax sessiliflorus* fruit extract on the antibacterial activity of porcine alveolar 3D4/31 macrophages via nuclear factor kappa B1 and lipid metabolism regulation

**DOI:** 10.5713/ajas.18.0874

**Published:** 2019-03-07

**Authors:** Eunmi Hwang, Gye Won Kim, Ki Duk Song, Hak-Kyo Lee, Sung-Jo Kim

**Affiliations:** 1 Division of Cosmetics and Biotechnology, College of Life and Health Sciences, Hoseo University, Baebang, Asan 31499, Korea; 2 Brewing Research Center, Academic Industry Cooperation, Hankyong National University, Anseong 17579, Korea; 3 Department of Animal Biotechnology, Chonbuk National University, Jeonju 54896, Korea

**Keywords:** Porcine, Feed Additives, *Acanthopanax sessiliflorus*, Macrophages, Nuclear Factor Kappa B (NF-κB), Immunity

## Abstract

**Objective:**

The demands for measures to improve disease resistance and productivity of livestock are increasing, as most countries prohibit the addition of antibiotics to feed. This study therefore aimed to uncover functional feed additives to help enhance livestock immunity and disease resistance, using *Acanthopanax sessiliflorus* fruit extract (ASF).

**Methods:**

ASF was extracted with 70% EtOH, and total polyphenolic and catechin contents were measured by the Folin-Ciocalteu and vanillin assay, respectively. The 3D4/31 porcine macrophage cells (MΦ) were activated by phorbol 12-myristate 13-acetate (PMA), and cell survival and growth rate were measured with or without ASF treatment. Flow-cytometric analysis determined the lysosomal activity, reactive oxygen species levels (ROS), and cell cycle distribution. Nuclear factor kappa B (NF-κB) and superoxide dismutase (SOD) protein expression levels were quantified by western blotting and densitometry analysis. Quantitative polymerase chain reaction was applied to measure the lipid metabolism-related genes expression level. Lastly, the antibacterial activity of 3D4/31 MΦ cells was evaluated by the colony forming unit assay.

**Results:**

ASF upregulated the cell viability and growth rate of 3D4/31 MΦ, with or without PMA activation. Moreover, lysosomal activity and intracellular ROS levels were increased after ASF exposure. In addition, the antioxidant enzyme SOD2 expression levels were proportionately increased with ROS levels. Both ASF and PMA treatment resulted in upregulation of NF-κB protein, tumor necrosis factor (TNF)α mRNA expression levels, lipid synthesis, and fatty acid oxidation metabolism. Interestingly, co-treatment of ASF with PMA resulted in recovery of NF-κB, TNFα, and lipid metabolism levels. Finally, ASF pretreatment enhanced the *in vitro* bactericidal activity of 3D4/31 MΦ against *Escherichia coli*.

**Conclusion:**

This study provides a novel insight into the regulation of NF-κB activity and lipid metabolism in MΦ, and we anticipate that ASF has the potential to be effective as a feed additive to enhance livestock immunity.

## INTRODUCTION

A common factor connecting maggots, alcohol, saliva, medicinal herbs, and amputation is that they have been employed as a method to resist infections by microorganisms, although most of these are folk remedies without scientifically proven effects. In recent days, maggot therapy has been found to effectively inhibit the growth of certain microorganisms and in the removal of dead skin cells [1]. Nonetheless, folk remedies have largely disappeared after the 1940s, since the advent of antibiotics eliminated concerns of progressing bacterial infections. The twenty-first century has seen the indiscriminate use of antibiotics in numerous fields including food manufacture, pharmaceutics, and farming, resulting in the production of millions of tons of antibiotics [2]. Furthermore, although antibiotic-resistant bacteria (ARB) were discovered in the 1960s, antibiotic usage has steadily increased, and currently, the ARB are a serious threat to public health. According to the Centers for Disease Control and Prevention (CDC), the annual mortality rates due to ARB have reached 23,000 in the United States (US), 25,000 in the European Union (EU), 38,000 in Thailand, and 58,000 in India [2,3]. As a solution to this problem, although efforts have been focused on developing new antibiotics, the outcomes have been unable to keep up with the continued increase of ARB [4].

The CDC has indicated that the primary cause of ARB is the inessential use of antibiotics in farming, being indiscriminately administered without a therapeutic purpose. Originally, antibiotics mixed in feed were provided to farms for preventing infectious diseases in livestock. However, once the growth-promoting effects of antibiotics were identified, the use of livestock antibiotics increased exponentially. Presently, it constitutes over 50% of the total antibiotics usage in most advanced countries, and accounting at over 80% in the US [5]. ARB in livestock can be transmitted to humans through food; furthermore, over 90% of livestock fed antibiotics are excreted, resulting in environmental pollution and ARB generation [3]. Hence, this study was undertaken to focus and investigate natural materials that improve the immunity and disease resistance of livestock, thereby laying the groundwork for the development of functional feed additives that will reduce indiscriminate antibiotic usage.

The mammalian immune system comprises of innate and adaptive immunity. Briefly, the porcine alveolar 3D4/31 macrophages (MΦ) are the innate immune cells that contribute to the lung immunity by removing the sources of infection, thereby performing a crucial function in disease resistance in livestock, especially in respiratory infectious diseases such as the porcine respiratory disease complex (PRDC) [6]. Nonetheless, no studies have actively investigated plant materials that may regulate livestock alveolar MΦ function. The MΦ are activated in the presence of cytokines or when toll-like receptors (TLR) recognize the endotoxin. The MΦ activation first induces acute morphological changes [7], at the same time enhancing the phagocytosis, inflammatory signaling, and lipid metabolism. These metabolic changes are controlled by nuclear factor kappa B (NF-κB), which is a transcription factor that induces the expression of proinflammatory genes such as tumor necrosis factor α (TNFα), interleukin (IL)-1β, IL-6, and IL-8 [8]. The enhanced lipid metabolism increases fatty acid synthesis, and fatty acid oxidation (FAO) a crucial factor for inducing alveolar MΦ activation [9,10]. Subsequently, the increased production of reactive nitrogen species (RNS) and reactive oxygen species (ROS) contribute to phagocytosis [11]. These metabolic changes enhance phagocytosis and help in removing the infection sources; however, the proliferation of activated MΦ is downregulated by cell cycle arrest, followed by rapid apoptosis [12].

Upregulated NF-κB signaling and lipid metabolism are essential for MΦ activation, although excessive enhancement may result in hyperinflammatory response and apoptosis [13]. Thus, for the effective improvement of innate immunity based on alveolar MΦ, the phagocytic activity needs to be enhanced, while suppressing the excessive lipid metabolism and hyperinflammatory response. In this study, we aimed to discover natural materials that enhance phagocytic activity while preventing cell death and inflammatory response. *Acanthopanax sessiliflorus* (*A. sessiliflorus*) grows in Korea, China, and Japan; the roots and rinds of *Acanthopanax* species have long been used in traditional medicines. In the present day, this substance provides health supplements due to its scientifically proven diverse effects on immunity; however, very few researches have studied the fruits [14,15], and there is no study yet on the effect of *A. sessiliflorus* fruits on porcine alveolar MΦ. Therefore, this study was undertaken to verify the effect of *A. sessiliflorus* fruit extract (ASF) on 3D4/31 MΦ, and confirm that exposure to ASF increases the survival rate and phagocytic activity. As the first attempt at investigating the effects of ASF on porcine MΦ, the findings of this study suggest new strategies and possibilities regarding the development of natural feed additive materials that are useful in enhancing porcine immunity, thereby reducing the use of antibiotics.

## MATERIALS AND METHODS

### Preparation of *A. sessiliflorus* fruit extract

The fruits of *A. sessiliflorus* were obtained in November 2014 from the Jeongseon Myungju Co.Ltd., Jeongseon, Korea. The air-dried fruits of *A. sessiliflorus* (100 g) were powdered and extracted three times for 24 h with 1 L of aqueous 70% EtOH at room temperature. The ASF was used after concentration *in vacuo*.

### Measurement of total phenolic and catechin contents

Total phenolic and catechin contents of ASF were measured by the Folin–Ciocalteu method and vanillin–catechin assay, respectively, as described previously [16]. The results are expressed in milligram of gallic acid or catechin equivalents per gram (dried weight) of ASF.

### Cell culture and reagents

Porcine 3D4/31 MΦ (ATCC-CRL-2844) were purchased from the American Type Culture Collection (ATCC, Manassas, VA, USA). The cells were cultured at 37°C in a CO_2_ incubator (Thermo Fisher Scientific, Waltham, MA, USA; 95% air and 5% CO_2_). The Roswell Park Memorial Institute 1640 medium (RPMI 1640; Corning, NY, USA) supplemented with 10% (v/v) thermally inactivated fetal bovine serum (Welgene, Gyeongsan, Korea) and 1% (v/v) of a penicillin-streptomycin solution (Welgene, Korea) was used for cell cultures.

### Cell viability and growth curve analysis

To assess the cell viability, 3D4/31 MΦ (5×10^3^ cells/well in 100 μL of media) were seeded in a 96-well cell culture plate (SPL Life Sciences, Pocheon, Korea). The cells were incubated for 12 h and treated with vehicle (distilled water; DW) or ASF (1.2, 12, and 120 μg/mL) for 24 h. Phorbol 12-myristate 13-acetate (PMA, 2 nM; Sigma-Aldrich, St. Louis, MO, USA) was then added for activation of MΦ. The final concentration of 10% (v/v) WST-1 reagent (EZ Cytox Cell Viability Assay Kit; DoGenBio, Seoul, Korea) was administered, followed by incubation for 2 h. The cell viability (optical density [OD] at 450 nm) was measured on a microplate reader (Sunrise, Tecan, Männedorf, Switzerland) and normalized to the vehicle (without PMA). For the growth curve analysis, 3D4/31 MΦ (5×10^4^ cells) were seeded in a 60 mm cell culture dish (SPL Life Sciences) and incubated for 12 h. ASF (120 μg/mL) was added to the cells and further incubated either with or without 2 nM PMA, and vehicle (DW). The medium was replenished and cells were treated again every 24 h, for 3 days. Growth curve was assessed by subjecting the cells to staining with 0.4% (w/v) trypan blue dye (Thermo Fisher Scientific, USA) and counting the cell number on a hemocytometer, for 3 days at every 24 h interval.

### Cell cycle analysis

3D4/31 MΦ (10^6^ cells) were seeded in a 60 mm cell culture dish and incubated for 12 h. The cells were treated with ASF (120 μg/mL) with or without PMA (2 nM) for 24 h, harvested, washed once in phosphate-buffered saline (PBS, pH 7.4), and resuspended in ice-cold ethanol, followed by 1 h incubation at −80°C for fixing and permeabilization. The fixed cells were again washed with PBS once, after which 175 μL of PBS with 5 μL of 10 mg/mL RNase A (R6513; Sigma-Aldrich, USA) was added and incubated for 30 min at 25°C, followed by staining with 500 μg/mL propidium iodide (PI; P-4170, Sigma-Aldrich, USA) for 10 min at 4°C in the dark. PI fluorescence intensity (cell cycle progression) was measured using the Guava EasyCyte (Millipore, Temecula, CA, USA) and FlowJo, version 10.0.7.2 (TreeStar, Ashland, OR, USA).

### Flow-cytometric analysis

3D4/31 MΦ (10^6^ cells) were seeded in a 60 mm cell culture dish and incubated for 12 h. The cells were treated with ASF (120 μg/mL) with or without PMA (2 nM) for 12 h. To measure the autophagic activity, the acidic vesicular organelle indicator acridine orange (AO; Sigma-Aldrich, USA) was administered at a final concentration of 1 μg/mL and incubated for 30 min. The treated cells were harvested and washed twice with PBS. The 3D4/31 MΦ ROS levels were measured using the ROS indicator dichlorofluorescein diacetate (DCFDA, 35845, Sigma-Aldrich, USA). DCFDA was administered at a final concentration of 10 μM, followed by incubation for 30 min. The cells were harvested and washed once with PBS. Autophagic activity and ROS levels were then measured by Guava EasyCyte. AO-positive cells and DCF-MFI (median fluorescence intensity) were evaluated in the FlowJo software, version 10.0.7.2.

### Western blot analysis

To measure the protein expression levels of NF-κB1 and superoxide dismutase 2 (SOD2) in 3D4/31 MΦ, Western blot was performed as described previously [16]. Briefly, total protein samples were extracted from 3D4/31 MΦ treated with ASF (120 μg/mL) with or without PMA (2 nM) for 12 h; 50 μg of protein was loaded per lane on an electrophoretic gel. Resolved proteins were then probed with anti-NF-κB1 (Santa Cruz Biotechnology, Santa Cruz, CA, USA), anti-SOD2 (Cusabio Technology, Houston, TX, USA), and anti-β-actin antibodies (Santa Cruz Biotechnology, USA). Relative protein expression levels were determined by densitometric analysis in ImageJ. At least three separate experiments were conducted for each protein.

### Real-time polymerase chain reaction

Real-time–polymerase chain reaction (RT-PCR) was carried out as described before [17]. Briefly, 3D4/31 MΦ (10^6^ cells) were seeded in a 60 mm dish and incubated for 12 h. The cells were treated with ASF (120 μg/mL) with or without PMA (2 nM) for 24 h. Total RNA was isolated using the TRIzol Reagent (Thermo Fisher Scientific, USA), and complementary DNA (cDNA) was synthesized by means of Moloney murine leukemia virus reverse transcriptase (Enzynomics, Daejeon, Korea). Next, 10 ng of cDNA was used for RT-PCR to determine the mRNA expression levels. Primer sequences are described in Table 1. Relative mRNA expression levels were analyzed by the 2^–ΔΔCt^ method.

### *In vitro* bacterial killing assay (colony-forming unit assay)

3D4/31 MΦ were treated with ASF (120 μg/mL) or vehicle (DW) for 24 h. *Escherichia coli* (*E. coli*) DH5α was cultured in the Luria-Bertani (LB) medium (Becton Dickinson, Franklin Lakes, NJ, USA) at 37°C and 200 rpm in a shaking incubator for 12 h, with additional incubation in fresh medium for 90 min. 3D4/31 MΦ and *E. coli* DH5α were harvested and washed twice with ice-cold Hank’s balanced salt solution (HBSS). 3D4/31 MΦ (10^6^ cells) and *E. coli* DH5α (2.5×10^6^ cells) were mixed in 1 mL of HBSS supplemented with 5% (w/w) porcine serum, followed by incubation at 37°C and 170 rpm in a shaking incubator for 2 h. For the MΦ killing assay, 100 μL of the cell mixture was collected at 15 min intervals, starting at 1 h after the incubation initiation. The collected cell mixtures were centrifuged at 1,500 rpm (213.81 g-forces) and 4°C for 1 min, washed with HBSS two times, and resuspended in 100 μL of DW followed by 5 min incubation at 25°C for disruption of MΦ. These disrupted cell mixtures were centrifuged at 1,500 rpm (213.81 g-forces) and 4°C for 5 min; 10 μL of the resultant supernatant was mixed with 90 μL of DW and spread on LB Agar plates. Colony-forming units (CFUs) were quantified after incubation for 12 h at 37°C. Representative LB Agar plate images were acquired with iPhone 6 (Apple Inc, Cupertino, CA, USA) and processed in Photoshop CC 2017 software (Adobe Systems, San Jose, CA, USA).

### Statistical analysis

All the data are obtained from at least three independent experiments. The data are expressed as the mean±standard deviation. Analysis was performed by GraphPad PRISM 7 (GraphPad Software, San Diego, CA, USA) and Microsoft Excel (Office 365, Microsoft, Redmond, WA, USA). Two-tailed Student’s *t*-test was used for measuring the p-value and significance of the data. Data are considered statistically significant when the p-value is <0.05.

## RESULTS

### Total phenolics and catechin contents in *A. sessiliflorus* fruit extract

For identifying the active ingredient of ASF, we determined the total phenolic and catechin contents. Our results showed that 1 g (dried weigh) of ASF contains 804.45±16.47 mg of total phenolics and 5.36±0.33 mg of catechin (Table 2). Plants produce unique secondary metabolites, depending on numerous surrounding factors, for adaptation or resistance to the environmental conditions [18]. Most of the polyphenols are secondary metabolites derived from natural plants; polyphenols are compounds with two or more phenol rings in their molecule, and are known to exert an antioxidant effect [19]. Furthermore, some polyphenols have anticancer, anti-inflammatory, and other beneficial biologic effects [20]. The well-known polyphenol catechin is composed of two benzene rings with a dihydropyran heterocycle. Several types of catechin enhance the phagocytosis activity of MΦ by controlling the cell death signaling [21]. Green tea leaves contain approximately 205.4±5.5 mg/g total catechin [22]; this it ~40-fold higher than that in ASF. However, *Panax ginseng* root, known as an effective immunostimulant, is reported to contain total phenolics at 32.4±0.1 μg/g, indicating that ASF contains approximately 24,800-fold more phenolics than *Panax ginseng* [23]. Our results therefore indicate that the phagocytosis-enhancing effect of ASF may not be caused by catechin, but may due to the large amounts of other polyphenols present.

### *A. sessiliflorus* fruit extract upregulates the cell survival rate

**Figure 1:**
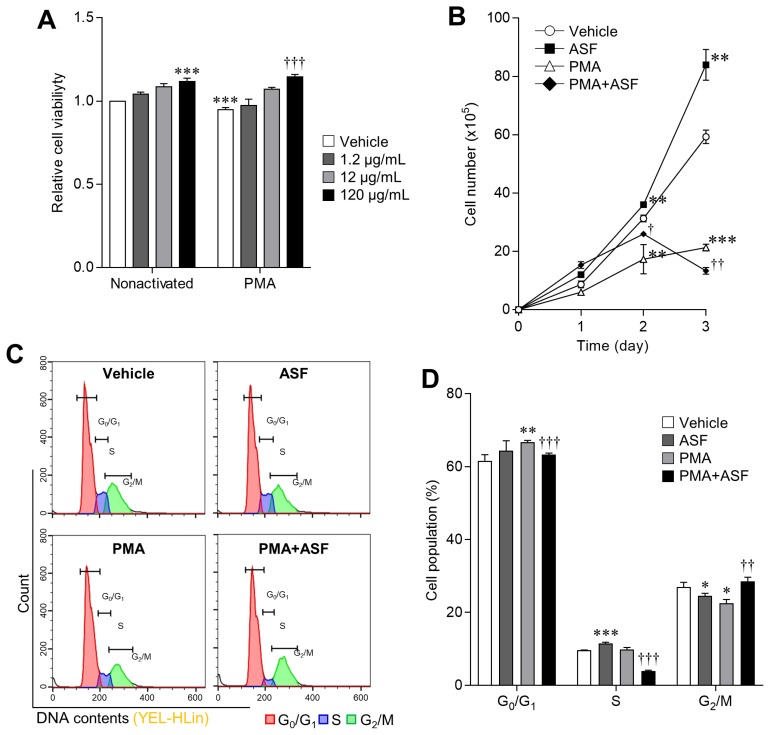
Upregulation effect of ASF on 3D4/31 MΦ cell viability and proliferation via cell cycle regulation. (A) WST-1 cell viability assay of ASF-treated 3D4/31 MΦ, with or without PMA for 24 h. (B) Growth curve analysis of 3D4/31 MΦ with ASF (120 μg/mL) or PMA (2 nM) treatment. (C) Representative flow-cytometric profiles of 3D4/31 MΦ with the PI dye. (D) Cell cycle populations of 3D4/31 MΦ treated with ASF (120 μg/mL) or PMA (2 nM) for 12 h. ASF, *Acanthopanax sessiliflorus* fruit extract; MΦ, macrophage; PMA, phorbol 12-myristate 13-acetate; PI, propidium iodide. Data represent the mean±standard deviation (n = 3); * p<0.01, ** p<0.01, *** p<0.001 vs vehicle; ^†^ p<0.01, ^††^ p<0.01, ^†††^ p<0.01 vs PMA treated group.

Activated MΦ have reduced viability and proliferation, and eventually undergo apoptosis [12]. Therefore, upregulation of viability and proliferation is important for enhancing MΦ activity. We evaluated whether ASF upregulated the viability and proliferation of 3D4/31 MΦ, with or without PMA (Figure 1A, 1B). We observed that cell viability of ASF-treated 3D4/31 MΦ increased in a dose-dependent manner, with maximum increase of 11.73%±2.02% at 120 μg/mL. Addition of PMA reduced the viability by 5.53%±1.13% relative to the vehicle, and ASF upregulated it up to 21.56%±0.63% (Figure 1A, p<0.001). In addition, ASF (120 μg/mL, 72 h treatment duration) upregulated the 3D4/31 MΦ proliferation rate up to 41.57%±8.92% (day 3, p<0.01), and PMA treatment downregulated it by 64.04%±1.95% (day 3, p<0.001), compared to the vehicle control. Furthermore, ASF treatment inhibits the PMA-induced antiproliferative effect up to 155.56%±13.32% (vs PMA, p<0.001) on day 1, and 50% on day 2 (vs group PMA, p<0.01, Figure 1B).

MΦ are the key and loyal soldiers of innate immunity; bacteria-induced activation reduces their proliferation and compromises their survival as they focus on phagocytosis [24]. ASF increased the survival rate of both non-activated and PMA activated 3D4/31 MΦ. Increased cell viability indicates two possible effects of ASF: on cell proliferation (number of mitochondria) or on mitochondrial activity [25]. Besides, the upregulation of mitochondrial activity can further contribute to cell proliferation. This increase in survival rates indicates that ASF is able to extend the MΦ life, enabling them to remove more bacteria.

### *A. sessiliflorus* fruit extract inhibits phorbol 12-myristate 13-acetate-induced cell cycle arrest

MΦ activation triggers cell cycle inhibition, consequent to a suppressed proliferation rate [24]. However, exposure to ASF upregulates the viability and proliferation of activated 3D4/31 MΦ (Figure 1A and 1B). To determine whether ASF has an effect on cell cycle regulation–based proliferation, we analyzed the cell cycle by PI staining, and confirmed that ASF inhibits the PMA-induced cell cycle arrest in 3D4/31 MΦ (Figure 1C and 1D, and Table 3). PMA treatment induces the cell cycle arrest in the G_0_/G_1_ phases; the proportion of PMA-treated cells in the G_0_/G_1_ phases increased up to 8.33%± 0.75% (vs vehicle, p<0.01), whereas ASF reduced this increase to 3.78%±0.68% (vs PMA, p<0.001). In addition, PMA treatment results in a decreased proportion of cells in the G_2_/M phases by 16.34%±3.81% (vs vehicle p<0.05), and exposure to ASF increased this proportion up to 26.48%±5.01% (vs PMA, p<0.01). However, in the absence of PMA, ASF induces cell cycle arrest in the G_0_/G_1_ phases. Cell cycle regulation depends on the activity of cyclin-dependent kinases (CDKs) and Cyclins. MΦ activation reduces proliferation via the regulation of cyclin D1 and p21 expression. Cyclin D1 is downregulated by MΦ activation, resulting in downregulation of the G1 phase [26], upregulation of p21, and inhibition of the binding of cyclin E to CDK2, followed by inhibition of cell cycle progression from G_1_ to S phase and subsequent apoptosis; however, cell cycle arrest by interferon γ suppresses the apoptosis [12]. Thus, the cell cycle arrest in MΦ may lower or increase the cell survival rate, depending on the induction method. Our results indicate that ASF upregulates the cell survival rates through different cell cycle–regulatory actions in nonactivated and activated 3D4/31 MΦ.

### *A. sessiliflorus* fruit extract upregulates lysosomal activity and reactive oxygen species production

**Figure 2:**
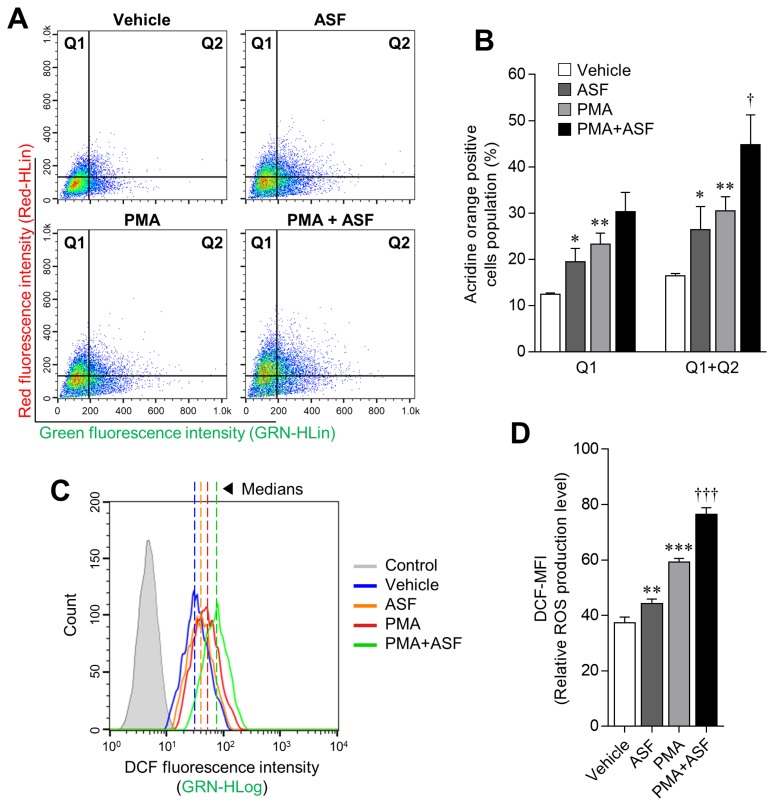
Boosting effect of ASF on the lysosomal activity of 3D4/31 MΦ. (A, C) Representative flow-cytometric profiles of 3D4/31 MΦ stained with acridine orange or the DCFDA dye. (B) Acridine orange–positive cell (Q1 and Q2) population of 3D4/31 MΦ treated with ASF or PMA for 12 h. (D) Relative ROS production levels (DCF-MFI) of 3D4/31 MΦ treated with ASF or PMA for 12 h. ASF, *Acanthopanax sessiliflorus* fruit extract; MΦ, macrophage; DCFDA, dichlorofluorescein diacetate; PMA, phorbol 12-myristate 13-acetate; ROS, reactive oxygen species. Data represent the mean±standard deviation (n = 3); * p<0.01, ** p<0.01, *** p<0.001 vs vehicle; ^†^ p<0.01, ^†††^ p<0.01 vs PMA treated group.

The progression of phagocytosis involves several steps, including ingestion of microbes and digestion of ingested microbes by the formation of phagolysosomes [27]. Digestion is achieved by a lysosome that contains a digestive enzyme with high levels of RNS and ROS. Hence, lysosomal activities play an important role in phagocytosis [28]. In this study, we confirmed that ASF and PMA treatment upregulate the lysosomal activity in 3D4/31 MΦ by 60.76%±30.67% (p<0.05) and 85.73%± 18.76% (p<0.01), respectively, relative to the vehicle (Figure 2A, 2B; Q1+Q2). ROS levels were also increased up to 18.36% ±4.35% by ASF treatment, and 58.38%±3.12% by PMA. Moreover, cotreatment with ASF-PMA upregulated the lysosomal activity and ROS levels up to 172.63%±39.61% and 104.55% ±3.95%, respectively, in comparison with the vehicle.

In normal tissue cells, ROS are mainly produced by mitochondrial metabolism; low levels of ROS play various signaling roles, whereas high concentrations are known to imduce inflammation and apoptosis in cells [29]. Most plant-based research is therefore aimed at reducing ROS levels to promote health [30]. However, ROS production is essential for innate immunity, as represented by bacterial phagocytosis. Activation of MΦ causes mitochondria to migrate into phagosomes and subsequent mitochondrial ROS (mROS) production. Although cellular ROS may be cytotoxic, the increase of mROS in MΦ is essential for phagocytosis [31]. Hence, the ROS-inducing effect of ASF in 3D4/31 MΦ may also have a positive influence on phagocytosis. In addition, ASF increases the survival rate and lysosomal activity of 3D4/31 MΦ. Taken together, these results indicate that ASF effectively increases the phagocytic activity of 3D4/31 MΦ.

### *A. sessiliflorus* fruit extract regulates MΦ activation and inflammatory signaling

**Figure 3:**
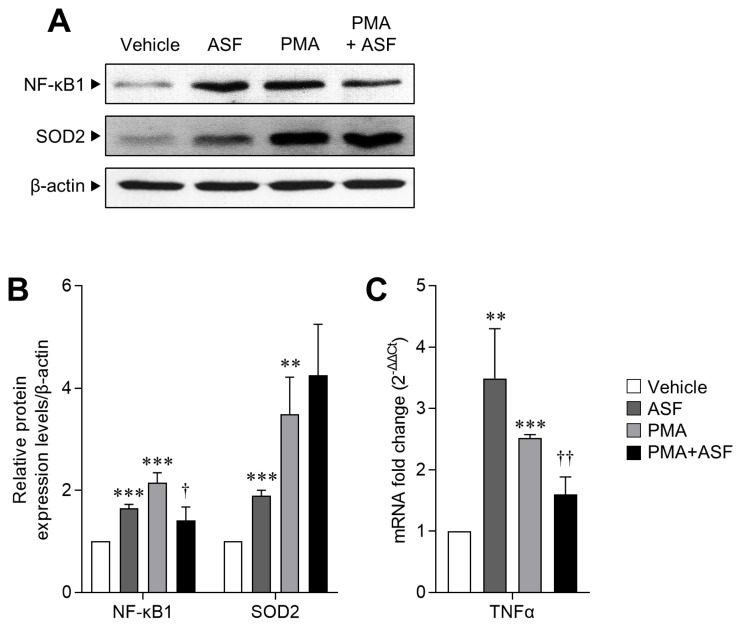
The regulatory effect of ASF on macrophage activation and inflammatory signaling of 3D4/31 MΦ. (A) Western blot analyses of NF-κB1, SOD2, and β-actin of 3D4/31 MΦ treated with ASF or PMA for 12 h. (B) Relative NF-κB1 and SOD2 protein expression levels of 3D4/31 MΦ. (C) TNFα mRNA fold change in 3D4/31 MΦ treated with ASF or PMA for 12 h. ASF, *Acanthopanax sessiliflorus* fruit extract; MΦ, macrophage; NF-κB1, nuclear factor kappa B; SOD 2, superoxide dismutase 2; PMA, phorbol 12-myristate 13-acetate; TNFα, tumor necrosis factor α. Data represent the mean±standard deviation (n = 3); ** p<0.01, *** p<0.001 vs vehicle; ^††^ p<0.01 vs PMA treated group.

Since ASF treatment results in upregulation of the cell survival rate and ROS levels in activated 3D4/31 MΦ, we next evaluated the expression levels of transcription factor NF-κB1 (as an MΦ activation and inflammation marker) and SOD2 (a key antioxidant enzyme of ROS redox signaling) [32]. We observed that relative to the vehicle, NF-κB1 expression levels of 3D4/31 MΦ increased by up to 65.05%±7.42% after ASF exposure, and 114.94%±19.36% after PMA treatment (p< 0.001). However, ASF treatment inhibited the PMA-induced NF-κB1 expression by 34.73%±6.23% (p<0.05). Furthermore, SOD2 expression levels were increased by up to 89.4%±10.74% (p<0.001) after ASF treatment, 249.30%± 72.42% (p<0.01) after PMA treatment, and 325.45%±99.35% (p<0.01) by ASF-PMA cotreatment (Figure 3A, 3B), as compared to vehicle control.

In mammalian cells, ROS are mainly produced by the mitochondrial electron transport, and are completely neutralized by SOD and other antioxidant enzymes and molecules. SOD is the most powerful antioxidant enzyme in our body, and is further classified as SOD1, 2, and 3, depending on the iron atom and its location. SOD2 (MnSOD) acts in the mitochondria [33], and therefore increased SOD2 expression suggests that upregulated ROS levels induced by ASF may be due to the formation of mROS, which are required for phagocytic activity [31]. Moreover, ASF induces the NF-κB1 expression but inhibits the same when MΦ are activated by PMA. In addition, NF-κB and TNFα serve as positive feedback mediators to induce the expression of each other [34]. We confirmed a similar expression pattern of TNFα mRNA to NF-κB1; PMA treatment increased the TNFα mRNA expression up to 151.72% ±6.02% (vs vehicle, p<0.001), and ASF reduced it by 36.23%±13.11% (vs PMA, p<0.01, Figure 3C). This result indicates that ASF inhibits the inflammatory signaling in activated MΦ but does not inhibit the MΦ differentiation and activation. Therefore, ASF has the potential to improve MΦ activity without inflammation.

### Distinct lipid metabolism-regulatory effects of *A. sessiliflorus* fruit extract in MΦ

**Figure 4:**
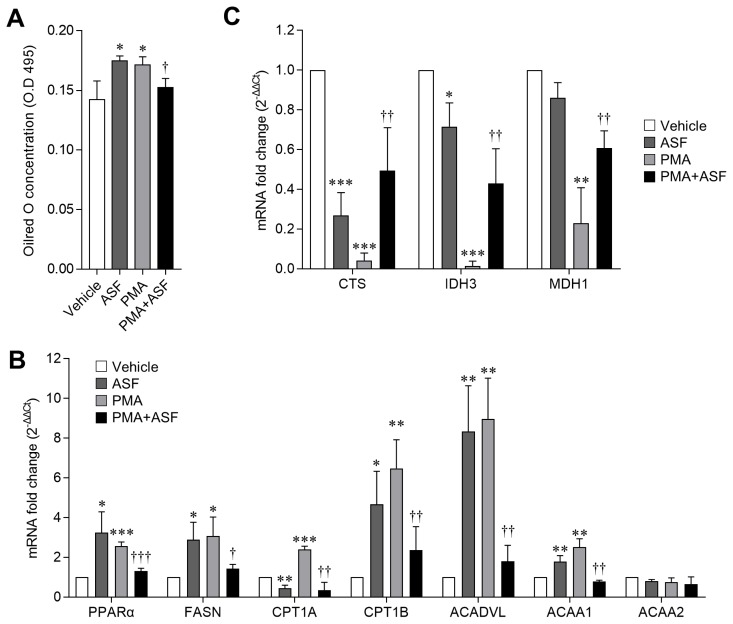
Regulatory effect of ASF on lipid metabolism of 3D4/31 MΦ. (A) Oil red O staining (lipid contents) in 3D4/31 MΦ treated with ASF or PMA for 24 h. (B, C) mRNA fold change of lipid synthesis and fatty acid oxidation (B) and Krebs cycle (C) related genes in 3D4/31 MΦ treated with ASF or PMA for 12 h. ASF, *Acanthopanax sessiliflorus* fruit extract; MΦ, macrophage; PMA, phorbol 12-myristate 13-acetate. Data represent the mean±standard deviation (n = 3); * p<0.01, ** p<0.01, *** p<0.001 vs vehicle; ^†^ p<0.01, ^††^ p<0.01, ^†††^ p<0.01 vs PMA treated group.

MΦ ingest lipoproteins from dying cells by phagocytosis, and eliminate their own intracellular lipids; in addition, infected alveolar MΦ reprogram their metabolism to be permissive for FAO thereby upregulating their lipid synthesis and FAO for effective immune response. However, excess lipids in MΦ result in the formation of foam cells or induce cell death, and FAO is essential for lipid-induced inflammation or lipotoxicity [13,35]. Moreover, since TLRs are also capable of recognizing lipoproteins and fatty acid, lipid-induced TLR signaling cascades upregulate the activation and expression of inflammatory genes (NF-κB, TNFα, and ILs) [36]. Hence, lipid and FAO balances are important for alveolar MΦ activity, survival, and inflammation. In this study, we confirmed that each ASF and PMA treatment upregulated the lipid metabolism, relative to the vehicle. ASF upregulated peroxisome proliferator-activated receptor alpha (PPARα) and fatty acid synthase (FASN) by up to 224.83%±104.39% and 189.22%±87.23%, respectively (p<0.05), whereas PMA upregulated PPARα and FASN by up to 156.27%±21.41% (p<0.001) and 206.68% ±95.9%, respectively (p<0.05). Conversely, ASF-PMA cotreatment reduced PPARα and FASN mRNA expression levels by half in comparison with the PMA treatment (vs PMA, p<0.05, Figure 4B). Accordingly, treatment with ASF and PMA upregulated the lipid contents (oil red O staining) by 22.64%±2.97% and 20.39% ±4.76%, respectively, as compared to the vehicle (p<0.05), and cotreatment of 3D4/31 MΦ with ASF-PMA cotreated reduced the lipid content by 11.05%±4.39% (p<0.05, Figure 4A) as compared to the PMA treated group.

Furthermore, increase in FAO related genes expression was observed after exposure to ASF or PMA, whereas ASF-PMA cotreatment downregulated the FAO genes expression levels. Especially, carnitine palmitoyltransferase 1 (CPT1) is strongly related to FAO and lipid-induced inflammation; CPT1 expression is essential for TLR induced inflammatory signaling that includes NF-κB and TNFα [35]. In addition, expression levels of Krebs cycle genes were inversely regulated by PMA; i.e., PMA inhibited the expression of 3D4/31 MΦ Krebs cycle genes. As expected, ASF-PMA cotreatment resulted in increased expression of Krebs cycle genes by 2 to 4 times, as compared to that of the PMA treatment group (Figure 4B, 4C). These lipid metabolism regulations by ASF indicate that ASF does not inhibit the activation of 3D4/31 MΦ. Since upregulation of lipid metabolism is essential for initiating MΦ activation, ASF therefore contributes to MΦ activation through lipid metabolism upregulation. However, the lipid metabolism and NF-κB expression (Figure 3B) of activated 3D4/31 MΦ are downregulated after ASF exposure, indicating inhibition of lipid-induced cell death, inflammation, and lipotoxicity.

### *A. sessiliflorus* fruit extract enhances the antibacterial activity of MΦ against *E. coli* DH5α

**Figure 5:**
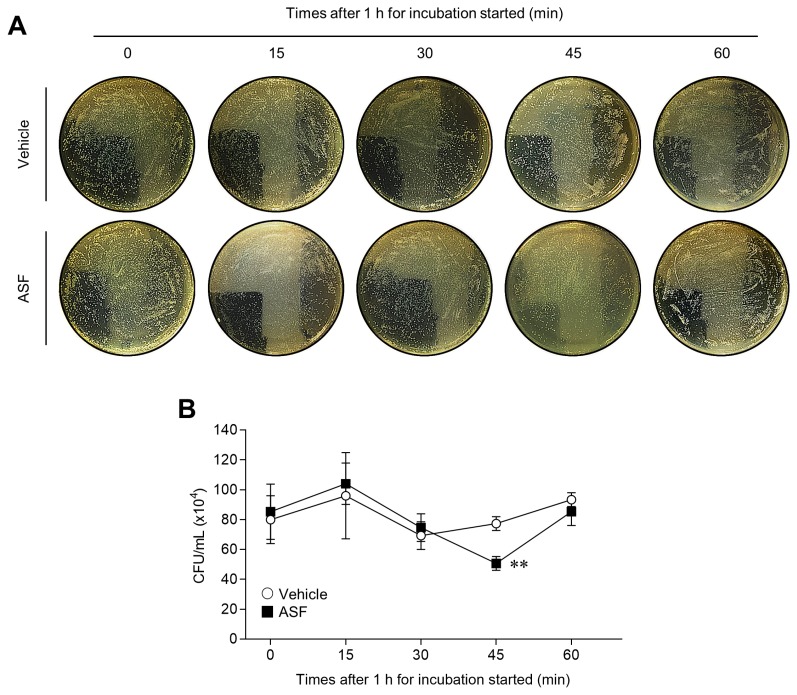
Antibacterial activity of 3D4/31 MΦ with ASF pretreatment. (A) CFU assay of 3D4/31 MΦ after ASF pretreatment for 24 h. (B) The CFU assay of ASF-pretreated 3D4/31 MΦ toward *Escherichia coli* DH5α. MΦ, macrophage; ASF, *Acanthopanax sessiliflorus* fruit extract; CFU, colony-forming unit. Data represent the mean±standard deviation (n = 3); ** p<0.01.

Since phagocytosis is the primary role of MΦ, any infection in the body results in MΦ activation in an attempt to eliminate the bacteria [37]. We therefore performed an *in vitro* bacterial killing assay (CFU assay) to examine the effect of ASF on the antibacterial activity of 3D4/31 MΦ toward *E. coli* DH5α. We quantified only the ingested *E. coli*, by washing the activated MΦ with HBSS and their subsequent disruption. We observed that the ASF-pretreated 3D4/31 MΦ resulted in higher mortality of bacteria than the vehicle-pretreated cells. The CFU was similar until 30 min for both treatment groups. However, CFU of the vehicle group at 45 min increased relative to that at 30 min, whereas CFU of the ASF group at 45 min decreased by 34.48%±5.97% (p<0.01) relative to the vehicle group (Figure 5A, 5B). After 45 min, an increase was observed in the number of bacterial CFU, indicating loss of phagocytic ability of the 3D4/31, subsequently followed by the imminent death of MΦ (total 105 min incubation) [38,39]. These results indicate that ASF increases the bacterial killing capacity by up to 40% by upregulating the cell survival rate (Figure 1) and lysosomal activity (Figure 2) in 3D4/31 MΦ.

## DISCUSSION

Respiratory diseases can be caused by infectious microorganisms as well as animal feed conditions, and these diseases have a devastating influence on agricultural workers and porcine health and productivity [40]. A greater problem regarding porcine health is the PRDC, a complex of various disorders and infections of the respiratory system, rather than a disease caused by a single causal infectious agent. PRDC frequently occurs in young pigs between the ages of 15 and 20 weeks, with morbidity as high as 40% and mortality 20%. Treatment of PRDC is challenging due to the late appearance of symptoms along with growth inhibition, and accompanying various infections [41]. Although antibiotics have been administered to prevent PRDC and promote growth, the recently discovered antibiotic resistance of the main pathogens of PRDC including *Streptococcus suis* and *Actinobacillus pleuropneumoniae* have posed challenges to the management of PRDC [42,43]. Among the *Actinobacillus pleuropneumoniae* isolates obtained from porcine farms in Australia, a country free from foot-and-mouth disease, 89% showed erythromycin resistance and 75% were tetracycline resistant [44]. Because the livestock antibiotics used for preventing infections and promoting growth were discovered to have strengthened the pathogenic bacteria with extended influence on human health, EU has banned the use of growth-promoting antibiotics (GPAs). However, this has resulted in an increased use of therapeutic antibiotics due to the increasing mortality of livestock and the decline in productivity, implying an urgent need to develop a method to replace GPAs [45]. Recent studies report that the growth-promoting effects of GPAs are induced by suppressed growth of microorganisms in the feed, inhibition of toxic microorganisms in the body of livestock, and enhanced anti-inflammatory activity and feed efficiency [46]. These data directed the focus of research to functional feed additives containing phytogenic materials (phytochemicals) as an alternative to GPA since they originate from natural plants, thereby causing lesser aversion or side effects in both livestock and humans while exerting high anti-inflammatory activity [47,48].

This study was therefore undertaken to examine whether ASF is a potential functional feed additive that could regulate livestock MΦ function and thereby contribute to reduced antibiotic usage. Our findings confirm that ASF significantly improves the antimicrobial activity and survival rate of MΦ by regulating the lysosomal activity, NF-κB expression, and lipid metabolism of porcine alveolar 3D4/31 MΦ. Besides the anti-inflammatory effect based on the reduced expression of proinflammatory cytokines [49,50], NF-κB inhibition suppresses the activity of TLR4 induced by lipopolysaccharide and phagocytosis of *E. coli*, given that NF-κB is an essential factor for MΦ activation [51]. Furthermore, MΦ activation results in reduced production of mitochondrial adenosine triphosphate (ATP) after the production of mROS [31], while increased lipid metabolism facilitates the absorption and synthesis of lipids, and PPARα promotes β-oxidation that utilizes fatty acids to produce ATP [52]. However, excessive intracellular accumulation of lipids that goes beyond MΦ functional capacity induces apoptosis [13]. Thus, NF-κB and lipid metabolism is a two-edged sword for MΦ activity and survival, and we found that ASF is able to regulate them effectively, such that it enhances the innate immune response during inactivation and inhibits inflammation and apoptosis upon activation by PMA. These effects of ASF suggest that a shift should be made from the conventional method of unconditionally inhibiting the regulatory factors of inflammation, such as NF-κB and TNFα in MΦ, to the use of independent methods of regulating NF-κB and lipid metabolism for the purpose of preventing infections and for post-infection treatment. The confirmed antimicrobial and anti-inflammatory activities of ASF and the findings of this study that demonstrate the alleviating effects of ASF on the porcine MΦ function and health, validates the high potential of ASF as a functional feed additive that can replace GPA. This study confirms that exposure to ASF enhances the porcine macrophage function. However, in addition to *E. coli*, there is a requirement to experiment with disease-causing bacteria that actually infect porcine (including PRDC related bacteria), and furthermore, *in vivo* testing is necessary to verify the efficacy of ASF in improving immunity, in small animal models such as mice. These extensive studies will help to further review and confirm the applicability of ASF to livestock, and enhance the possibility of development as an alternative to GPA and a functional feed additive.

## Figures and Tables

**Table 1:** Primer sequences for 3D4/31 MΦ

Gene	Sequences (5′ to 3′)
*TNFα*	F: CGTTGTAGCCAATGTCA
(tumor necrosis factor alpha)	R: TAGGAGACGGCGATGC
*PPARα*	F: TTGAACGACCAGGT CACGCT
(peroxisome proliferator-activated receptor)	R: GGAACTCGCGCGTGATGAAG
*FASN*	F: GTGGAGGTGCGCCAGATACT
(fatty acid synthase)	R: CCTCGTGGGATGTGGGAGTC
*GAPDH*	F: GTCGGAGTGAACGGATTTGGC
(glyceraldehyde 3-phosphate dehydrogenase)	R: ACTGTGCCGTGGAATTTGCC
*CPT1A*	F: CAAGATAGCGGCCGAAAAGC
(carnitine palmitoyltransferase 1a)	R: GATAATCGCCACGGCTCAGA
*CPT1B*	F: CCACTATGACCCGGAAGACG
(carnitine palmitoyltransferase 1b)	R: TTGAACGCGATGAGGGTGAA
*ACADVL*	F: GCGGTGAATCATGCTGCTAA
(acyl-CoA dehydrogenase, very long chain)	R: GTGGATCCCTGGTCCATGTT
*ACAA1*	F: TCGCCCAGTTTCTGAGTGAC
(acetyl-CoA acyltransferase 1)	R: CCACAAGCCATGCCAATGTC
*ACAA2*	F: TCGTGGGCTATTTTGCGTCT
(acetyl-CoA acyltransferase 2)	R: TCCTGCTTTCTTCAGTGCCC
*CTS*	F: GGGCACTGGGTGTATTAGCA
(citrate synthase)	R: TCATGGACTTGGGCCTCTCT
*IDH3*	F: CGCTGCAAAGATTGAGACCG
(isocitrate dehydrogenase 3)	R: TCTGAGCATTTTGCGTTGCC
*MDH1*	F: TGGTGTTCCTGATGATCTGCTC
(malate dehydrogenase1)	R: ACCCTTCAGCTCTGCAACAA

MΦ, macrophage; F, forward; R, reverse.

**Table 2:** Total phenolic and catechin content in *Acanthopanax sessiliflorus* fruit extract

Items	
Dried weight (mg/mL)	119±0.001
Catechin (mg/g)	5.36±0.33
Total phenolic (mg/g)	804.45±16.47

Data expressed as mean±standard deviation value of three replicates.

**Table 3:** Cell cycle population of ASF treated 3D4/31 macrophage

Items	Vehicle (%)	ASF (%)	PMA (%)	PMA+ASF (%)
G_0_/G_1_	61.60±1.68	64.33±2.77	66.73±0.46^b^	63.27±0.45^e^
S	9.60±0.10	11.50±0.35^c^	9.83±0.56	3.94±0.18^e^
G_2_/M	26.93±1.30	24.57±0.68^a^	22.53±1.03^a^	28.50±1.14^d^

Data expressed as mean±standard deviation value of three replicates.

ASF, *Acanthopanax sessiliflorus* fruit extract; PMA, phorbol 12-myristate 13-acetate.

Statistical significance was assessed with respect to the vehicle or PMA treated sample in the same phase (vs vehicle: ^a^ p<0.05, ^b^ p<0.01, ^c^ p<0.001; vs PMA treated: ^d^ p<0.01, ^e^ p<0.001).
